# Depressive Symptoms and Associated Factors in Female Students in Fukushima Four Years after the Fukushima Nuclear Power Plant Disaster

**DOI:** 10.3390/ijerph15112411

**Published:** 2018-10-30

**Authors:** Shinya Ito, Mie Sasaki, Satoko Okabe, Nobuhiro Konno, Aya Goto

**Affiliations:** 1 School of Nursing, Kitasato University, Kanagawa Prefecture 252-0329, Japan; 2 Faculty of Humanities, Saitama Gakuen University, Saitama Prefecture 333-0831, Japan; m.sasaki@saigaku.ac.jp; 3 Department of Food and Nutrition, Koriyama Women’s University, Fukushima Prefecture 963-8503, Japan; okabe@koriyama-kgc.ac.jp (S.O.); nkkonno@koriyama-kgc.ac.jp (N.K.); 4 Center for Integrated Science and Humanities, Fukushima Medical University, Fukushima Prefecture 960-1295, Japan; agoto@fmu.ac.jp

**Keywords:** depression, Fukushima nuclear accident, radiation, Future Parents Attitude Measure, women, students, reproductive health

## Abstract

Young women in their late teens and early 20s are at the highest risk for depression onset. The present study aimed to assess depressive symptoms among female college students in Fukushima. More specifically, it aimed to clarify factors predicting possible symptom profiles, with an emphasis on determining how nuclear radiation risks affect the reporting of depression symptoms. A cross-sectional survey was conducted of 310 female students at a college in the Fukushima prefecture, Japan, in December 2015, and 288 participants submitted valid questionnaires. In total, 222 (77.1%) participants lived in Fukushima at the time of the Great East Japan Earthquake. The measures included the World Health Organization-Five Well-Being Index, the Fukushima Future Parents Attitude Measure, and risk perception of radiation health effects. A total of 46.5% of participants reported depressive symptoms. Path analysis revealed that higher radiation risk perceptions and reduced efficacy with reproduction related to a decline in self-esteem and self-efficacy, which was subsequently associated with increased depressive symptoms. These findings highlight the importance of radiation education among children and young adults, both after a nuclear accident and during disaster preparation, particularly in the context of reproductive and mental health.

## 1. Introduction

The Great East Japan Earthquake occurred on 11 March 2011. A subsequent tsunami hit the Tokyo Electric Power Company’s Fukushima Daiichi Nuclear Power Plant, causing leakage of radioactive material from the plant. Exposure to both natural and man-made disasters is associated with long-lasting psychological problems [1,2,3,4,5]. Using data derived from the Pregnancy and Birth Survey from the Fukushima Health Management Survey (FHMS) targeting mothers (median age = 30 years) with infants, it was found that 28% of the sample of 2262 women screened positive for depression in 2011 [1]. In a survey of 180,604 evacuees of the Fukushima nuclear disaster in 2012, perception of radiation risks was associated with psychological distress [5].

In the offspring of people exposed to radiation, increased cancer incidence related to hereditary effects has not been reliably demonstrated for any level of exposure [6,7]. However, the Pregnancy and Birth Survey of FHMS, a prefecture-wide cohort study conducted between 2011 and 2013, revealed that 13.0–29.8% of pregnant women were concerned about the “radiation effect on fetus and infant” [8]. Young women in the Fukushima prefecture worry that others might view them negatively based on assumptions about radiation effects on future pregnancies or genetic inheritance [9]. The self-awareness of the young women could be regarded as a “self-stigma” induced by the public stigma related to radioactive contamination. Self-stigma is defined as “the devaluation of the self as a result of internalizing a stigmatized identity associated with the negative stereotypes about one’s social group” [10]. Previous research has shown that the experience of self-stigma is associated with poorer self-esteem [11,12], reduced self-efficacy [11,12], and decreased quality of life (QOL) [11]. Self-esteem and self-efficacy in some people with psychiatric disorders, including depression, were affected by self-stigma regarding mental illness. For example, Corrigan et al. [12] used the Self-Stigma of Mental Illness Scale and instruments that measured self-esteem, self-efficacy, and depression to examine these issues in 60 people with psychiatric disabilities. The results provided evidence supporting their four-level model of self-stigma: awareness of stereotypes, agreement with stereotypes, applying stereotypes to oneself, and diminished self-esteem and self-efficacy. Self-esteem is defined as a person’s overall evaluation of his or her worthiness as a human being [13]. The Rosenberg Self-Esteem Scale (RSES) [14] is the most widely used instrument for the measurement of global self-esteem. Self-efficacy is defined as people’s beliefs about their capabilities to produce designated levels of performance that exercise influence over events that affect their lives [15,16]. Some studies have demonstrated associations between self-stigma and low self-esteem and self-efficacy [11,12], and others showed that levels of self-esteem and self-efficacy were related to the prevalence of depression [17,18]. We constructed a model whereby we hypothesized that “perception of radiation risk” influenced reproductive confidence (i.e., being able to deliver a baby safely), reproductive confidence influenced self-esteem and self-efficacy, and self-esteem and self-efficacy influenced depressive symptoms.

The 12-month prevalence rate for major depressive disorder according to diagnostic criteria based on the Diagnostic and Statistical Manual of Mental Disorders, Fifth Edition (DSM-5) was roughly 7% [19]. This prevalence rate is higher for women (lifetime rate = 23.4%) than it is for men (5.7%) [20,21]. Depression in adolescence is a major risk factor for suicide [22], which has been identified as a leading cause of death in this age group [22,23]. However, enhanced self-efficacy [24], self-esteem [24], and empowerment [12] are associated with diminished rates of depression. Children who began junior high school around the time of the disaster have since graduated from high school and started careers and families. Although the prevalence of major depressive disorder in younger age groups (18–29 years) is higher relative to that observed in other age groups, the relationship between depressive symptoms and radiation risk perception has not been clarified among female students from Fukushima. This issue is especially pertinent, as these women experienced a major disaster during their late adolescence or early adulthood and reside in a region exposed to wide-reaching radiation contamination. Relatedly, a study conducted between 1999 and 2002, after the Chernobyl accident, showed a depression rate of 13% among 115,191 adolescents, and 77% of the diagnostic group were girls [3]. A similar investigation is needed in Fukushima, to plan appropriate support strategies for the affected populace.

We hypothesized that the proportion of female students in Fukushima reporting depressive symptoms would be higher relative to those of female students in other areas. Further, we hypothesized that, after radiation exposure, radiation risk perception and anxiety related to pregnancy and childbirth would be related to the reporting of depressive symptoms. The aim of the present study was to determine the frequency with which female college students reported depressive symptoms in Fukushima. In addition, we sought to clarify the factors underlying symptom profiles, with a focus on radiation risk perception.

## 2. Materials and Methods

### 2.1. Study Design and Participants

A cross-sectional survey was distributed to 310 female students at a college in the Fukushima prefecture, Japan, in December 2015. As random sampling of participants and colleges was difficult, a college with a high proportion of female students from Fukushima was selected by the authors. Prior to the study period, we obtained university approval and held preparatory meetings with teachers. The teacher of each class distributed the questionnaires and collected them from participants once complete. The participants were all female students at all levels and specialized in nutrition. Potential participants were excluded on the following grounds: currently married (n = 3); provided the same response to all questions (n = 1); missing data in the World Health Organization-Five Well-Being Index (WHO-5; n = 1) [25]; a score of 9 out of 10 on the Japanese Social Desirability Scale (n = 9) [26]; or, missing Japanese Social Desirability Scale values (n = 8) [26]. As depressive symptoms were a main outcome in this study, participants with missing data in the WHO-5 were excluded. A social desirability effect might interfere with the appropriate response to items about radiation exposure. Therefore, we excluded data for participants who scored 9 out of 10 on the Japanese Social Desirability Scale or did not respond to this measure (the Japanese Social Desirability Scale [26] consists of 10 items rated on a 2-point Likert scale). Examples of items include “If I could get into a movie without paying and be sure I was not seen, I would probably do it” and “I have almost never felt the urge to tell someone off”. Ultimately, 288 participants were included in the final analyses.

### 2.2. Instruments

Concerning demographics, we evaluated age, living arrangements, and residence prefecture at the time of the Great East Japan Earthquake. The WHO-5 [25], which is a self-administered 5-item scale, was used to assess depressive symptoms. Each item measures the degree of positive well-being experienced during the preceding 2 weeks, via a 6-point Likert scale ranging from 0 to 5, with higher scores indicating an increased sense of well-being [21]. Participants who answer 0 or 1 for any of the 5 items or have a total score below 13 are considered to experience depressive symptoms [27].

Additional measures included the Fukushima Future Parents Attitude Measure (FPAM) [28], the RSES [14,29], the General Self-Efficacy Scale (GSES) [30], and questions relating to the perception of risk of radiation health effects [31,32]. The FPAM was used to measure future mothers’ attitudes in relation to pregnancy and childbirth after being exposed to radiation. It comprises 2 factors: “Caring for a baby” (3 items) and “Giving birth to a baby” (3 items). “Caring for a baby” includes “I look forward to caring for the baby”, “I believe that I will enjoy caring for the baby”, and “I feel that babies are not much fun to care for”. “Giving birth to a baby” includes “I am worried that the baby might have problems”, “I am confident that I will have a normal childbirth”, and “I think my labor and delivery will progress normally”. The items are measured on a 4-point Likert scale, graded from 1 (Strongly agree) to 4 (Strongly disagree). The scores for “Caring for a baby” and “Giving birth to a baby” range from 3 to 12, with higher scores representing higher expectation of future pregnancy or childbirth. Before responding to the items, participants are provided with the following instruction: “Please answer the following questions. Please assume that you will live and raise a family in Fukushima prefecture”. The details of the questionnaire (including the Japanese version) have been reported previously [28]. The RSES defines self-esteem as a global concept of the self and a sense of worth or value, not as the possession or accumulation of specific qualities or abilities [29]. It is a 10-item scale, and responses are measured via a 4-point Likert scale ranging from 10 (strongly disagree) to 40 (strongly agree), with higher scores representing higher self-esteem. The reliability and validity of the Japanese version of the RSES has been documented [29]. The GSES, which is a 16-item dichotomous (yes = 1/no = 0) scale, was used to measure global self-efficacy. Scores range from 0 to 16, with higher scores representing higher self-efficacy. The reliability and validity of the Japanese version of the GSES has been reported [30]. We evaluated risk perception regarding radiation health effects using a 2-item screening measure from a mental health and lifestyle screening tool, via the FHMS [31,32]. The 2 items were “What do you think is the likelihood of damage to your health (e.g., cancer onset) in later life as a result of your current level of radiation exposure?” (delayed effects) and “What do you think is the likelihood that the health of your future (i.e., as yet unborn) children and grandchildren will be affected as a result of your current level of radiation exposure?” (genetic effects). Participants were asked to respond to each question using a 4-point Likert scale as follows: 1 = very unlikely, 2 = unlikely, 3 = likely, and 4 = very likely. Scores range from 1 to 4, with higher scores representing greater subjective health effects. These questionnaire items have been used often in Japan since the 2011 disaster.

### 2.3. Statistical Analyses

**Figure 1:**
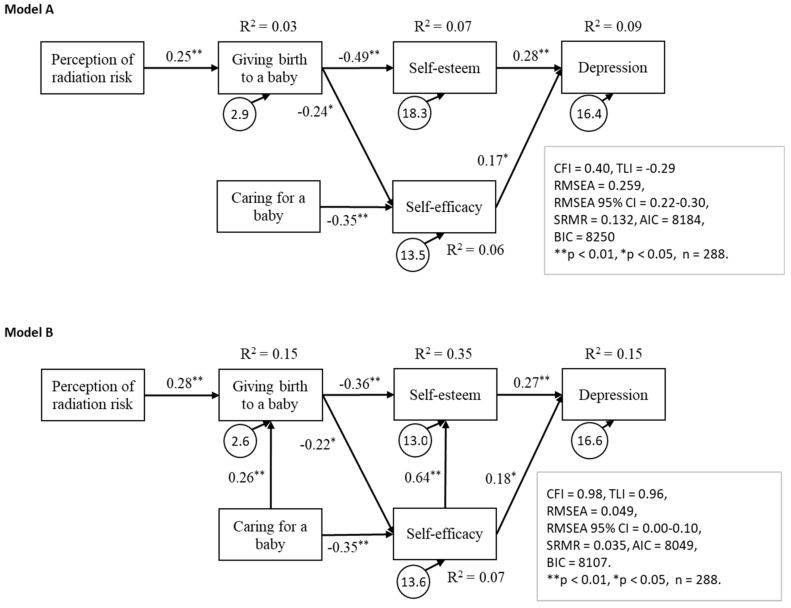
The pathway model for depressive symptoms.

To assess potential group differences in sociodemographic characteristics between participants with (“Positive” group) and without (“Negative”) depressive symptoms, we conducted chi-squared tests, Student’s t tests, and Mann-Whitney U tests. Conformity to linearity was checked graphically. To assess predictors of depressive symptoms, we conducted a multivariate logistic regression analysis via the forced entry method. We calculated odds ratios (ORs) and 95% confidence intervals (95% CIs). In the logistic regression analysis (Model 1), the dependent variable was the presence or absence of depressive symptoms, and the independent variables were living arrangements, age, prefecture (residence prefecture at the time of the Great East Japan Earthquake: Fukushima or other prefecture), and perception of radiation risk. Model 2 was created as multiple logistic regressions with adjustment for RSES and GSES scores. The independent variables in Model 2 were living arrangements, age, prefecture, and perception of radiation risk. In addition, we performed path analysis. In the first model (Figure 1, Model A), the endogenous variables were “Caring for a baby” and “Giving birth to a baby” in the FPAM; scores for the RSES, GSES, and WHO-5 were the exogenous variables and represented the perception of risk of radiation health effects. In a revised model (Figure 1, Model B), the endogenous variables were “Giving birth to a baby” and RSES, GSES and WHO-5 scores, and the exogenous variables were “Caring for a baby” and the perception of risk of radiation health effects. Five fit statistics were used to assess goodness of fit [33]: the comparative fit index (CFI), the Tucker-Lewis Index (TLI), root mean square error of approximation (RMSEA), RMSEA 90% CI, and the standardized root mean square residual (SRMR). The missing data were accommodated via multiple imputation method (imputed data set: M = 5) by SPSS and full information maximum likelihood by Mplus. Path analysis was performed using Mplus 3.0 (Muthén & Muthén, Los Angeles, CA, USA), and other statistical tests were conducted via SPSS (version 21.0; SPSS Inc., Chicago, IL, USA).

### 2.4. Ethical Considerations

The Fukushima Medical University ethics committee approved this study (No. 2462). The survey was anonymized using a bubble answer sheet and the separate distribution of the questionnaire and answer sheet. The survey aims were explained to all participants in a cover letter that was distributed with the questionnaire. The cover letter stated that students should return a blank questionnaire if they did not wish to participate. We also informed participants that the survey was anonymous and they would not suffer adverse consequences based on their responses or withdrawal of participation. Participants were considered to have provided consent by returning the survey.

## 3. Results

We received responses from all 310 participants, and 288 participants submitted valid responses (response rate = 92.9%). The data for participants with missing values in each measurement were excluded from the analysis. Participants’ characteristics, based on the presence and absence of depressive symptoms, are shown in Table 1 and Table 2. Depressive symptoms were reported by 134 participants (46.5%). However, depressive symptoms were not significantly associated with sociodemographic characteristics such as living arrangements, age, and prefecture (Table 1). Conversely, the “Caring for a baby” (*p* < 0.01), “Giving birth to a baby” (*p* < 0.05), self-esteem (*p* < 0.01), and self-efficacy (*p* < 0.05) scores were significantly associated with depressive symptoms.

The correlations between the variables are presented in Table 3, and those observed in the multivariate logistic regression analyses are presented in Table 4. In Model 1, “Caring for a baby” and “Giving birth to a baby” were significantly associated with depressive symptoms. In Model 2, age was significantly associated with depressive symptoms.

We performed path analysis to identify a causal model for depressive symptoms. We constructed a model in which “perception of radiation risk” influenced FPAM scores, FPAM scores influenced self-esteem and self-efficacy, and self-esteem and self-efficacy influenced depressive symptoms (Figure 1, Model A). The goodness of fit for Model A was unacceptable (n = 288, CFI = 0.40, TLI = −0.29, RMSEA = 0.259, RMSEA 95% CI = 0.22–0.30, SRMR = 0.132, AIC = 8184, BIC = 8250). Thus, in the revised model, the statistically nonsignificant paths were removed (Figure 1, Model B). As confidence in child rearing in a disaster area might be related to confidence in delivery, we added a path from “Caring for a baby” to “Giving birth to a baby”. The goodness of fit for the revised model was acceptable (n = 288, CFI = 0.98, TLI = 0.96, RMSEA = 0.049, RMSEA 95% CI = 0.00–0.10, SRMR = 0.035, AIC = 8049, BIC = 8).

## 4. Discussion

In total, 46.5% female college students from Fukushima reported depressive symptoms. Symptom reports were significantly associated with “Caring for a baby”, “Giving birth to a baby”, self-esteem, and self-efficacy. In contrast, living arrangements, place of residence, and perception of radiation risk were not significantly related to depressive symptoms. Based on the path analysis, perception of higher radiation risk was predictive of reduced reproductive confidence, which was associated with decline in self-esteem and self-efficacy, and ultimately associated with increased depressive symptoms. Although no clear epidemiological hereditary effects of radiation exposure in human beings have been reported [2,3], the perception of radiation risk was related to reproductive confidence in the present study. These findings suggest the importance of addressing young women’s educational needs regarding the health effects of radiation. Specifically, such care should focus on not only radiation issues but also women’s efficacy regarding their reproductive and mental health. In addition, only after a nuclear power incident would radiation education be meaningful for routine crisis management.

The proportion of female students reporting depressive symptoms in Fukushima was quite high. This was inconsistent with the findings of prior studies assessing mothers with infants in Fukushima, in which rates of depression ranged from 23.4% to 27.1% based on results obtained via a 2-item screening instrument [4]. The rates reported in the present study were much higher, even in comparison to previous reviews [34,35] and data obtained from young mothers at the time of the Fukushima nuclear accident [4,8]. A systematic review of research conducted between 1990 and 2010 to assess the prevalence of depression among female students reported depression rates with a weighted mean average of 29.6% [34]. A meta-analysis of 35 studies conducted in Iran from 1995 to 2012 showed a prevalence rate of 23% among female university students [35]. Our results suggest that providing mental health support over longer periods is important for female students in Fukushima.

According to the path analysis, perception of radiation risk did not directly influence depressive symptoms. Instead, the results showed an indirect relationship between these two variables, which is inconsistent with the findings of previous studies [5,36]. Among 8717 evacuees from the Fukushima nuclear disaster, the perception of radiation risk was directly associated with psychological distress [5] in 2012. During the first year following the accident, radiation-related information was lacking in detail; thus, mothers with infants in Fukushima felt particularly anxious regarding potential radiation effects [8]. Therefore, the perception of radiation risk was associated with psychological distress among evacuees who were mothers at the time of the accident. Conversely, as individuals are better able to obtain radiation-related information four years after the accident, the perception of radiation risk may be less directly linked to depressive symptoms. The results showing relationships between reproductive confidence (i.e., being able to deliver a baby safely), self-esteem, self-efficacy, and depressive symptoms are similar to those of previous studies [10,11,12]. However, the coefficient for the determination of depression was low. For current female university students, other issues (e.g., in relation to student life) may be more directly associated with mental health, and radiation risk perception may have a specialized association with depressive symptoms (e.g., through a variety of mediators including efficacy regarding reproductive health). As the study design was cross-sectional, causal relationships between variables are uncertain. However, indirect associations may be present within the combined influence of risk perception on reproductive and mental health. Interventions for self-esteem and self-efficacy might lead to improvements in depressive symptoms present after the accident.

Three notable methodological limitations were present in the current study. First, the study design was cross-sectional, and further studies are needed to clarify potential causal relationships between the variables assessed. Second, the research was conducted with a sample of women from a single college. In the Fukushima prefecture alone, there are 14,053 students (men = 9795, women = 4258) across eight colleges, including an additional 1711 students (men = 134, women = 1577) across five two-year colleges as of 2016. Thus, the small sample, relative to the broader population affected by the 2011 accident, might limit the generalizability of the present findings. In particular, it is unclear whether the prevalence rate for depression differs from those reported by previous studies, as poorer response rates tend to be associated with higher prevalence rates [34]. As the response rate in this study was high, we could interpret the results in terms of the college. However, the proportion of students reporting depressive symptoms in Fukushima should be considered with some caution. Third, our study did not evaluate additional variables that are likely to be associated with depression within a collegiate sample (i.e., stress regarding job searches, examinations, interpersonal relationships, etc.). For example, we did not examine stressors related to college life. In addition, stressors might differ between grades. Although age was significantly associated with depressive symptoms, stressors might be a confounding factor in this relationship. In addition, previous studies have suggested that positive emotions play a key role in recovery from depressive symptoms [37]. Thus, further studies are needed to clarify additional factors affecting depressive symptoms, above and beyond the residual effects of the Fukushima nuclear disaster.

## 5. Conclusions

Given the relatively high rate of depressive symptoms observed in the present sample, improving mental health support over the long term is imperative for women in Fukushima. Specifically, the provision of support should target not only the paucity of knowledge regarding radiation exposure risk but also anxiety related to both reproductive and mental health.

## Figures and Tables

**Table 1:** Participants’ sociodemographic characteristics.

		Depressive Symptoms *^1^				*p*-Value	Chi-Squared	φ or Cramer’s V
		Negative (n = 154)		Positive (n = 134)				
		n	%	n	%			
Living arrangement								
	Living alone	31	20.3	28	21.1	0.869	0.03	0.010
	Living together	122	79.7	105	78.9			
Age (years)								
	18	14	9.2	16	11.9	0.203	7.25	0.159
	19	48	31.4	38	28.4			
	20	43	28.1	44	32.8			
	21	27	17.6	29	21.6			
	22	19	12.4	6	4.5			
	≥23	2	1.3	1	0.7			
Residence Prefecture at the Great East Japan Earthquake								
	Fukushima prefecture	118	78.1	104	78.2	0.992	0.00	0.001
	Other prefectures	33	21.9	29	21.8			

*^1^ Participants in the positive group for depressive symptoms scored 1 or 0 for any of the 5 World Health Organization-Five Well-Being Index items or had an overall score of <13.

**Table 2:** Comparison of scale scores for depressive symptoms between the negative and positive groups.

Scale (Range of Score)		Cronbach’s Alpha Coefficient	Depressive Symptoms *^1^						*p*-Value	Effect Size d or r	Difference of 95% CI	
			Negative			Positive						
			n	M	SD	n	M	SD			Lower	Upper
The FPAM scale *^2^												
	Caring for a child (3–12)	0.86	154	5.3	2.1	134	6.1	2.4	0.004	0.36	−1.29	−0.24
	Giving birth to a baby (3–12)	0.65	154	6.6	1.7	134	7.0	1.8	0.041	0.29	−0.83	−0.02
Self-esteem (10–40)		0.71	154	24.2	4.3	134	22.7	4.5	0.003	0.36	0.55	2.60
Self-efficacy (0–16)		0.80	154	6.5	3.9	134	5.4	3.6	0.016	0.29	0.20	1.99
Perception of radiation risk (2-item score: 2–8)		—	154	4.8	1.3	134	4.6	1.4	0.161	0.15	−0.09	0.52
	Delayed effects (1–4) *^3^	—	154	2.0		134	2.0		0.100	0.10	—	—
	Genetic effects (1–4) *^3^	—	154	2.0		134	2.0		0.427	0.05	—	—

*^1^ Participants in the positive group for depressive symptoms scored 1 or 0 for any of the 5 World Health Organization-Five Well-Being Index items or had an overall score of <13. Number of missing data: FPAM = 5, self-esteem = 2, self-efficacy = 8. *^2^ FPAM = Fukushima Future Parents Attitude Measure. *^3^ We conducted a Mann-Whitney *U* test and calculated the median.

**Table 3:** Correlations between scales *^1^.

		1	2	3	4	5	6
	FPAM *^2^						
1	Caring for a child (3–12)	—	0.32 **	−0.22 **	−0.20 **	−0.23 **	−0.06
2	Giving birth to a baby (3–12)		—	−0.16 **	−0.22 **	−0.16 **	0.19 **
3	WHO-5 *^3^ (0–25)			—	0.36 **	0.29 **	0.03
4	Self-esteem (10–40)				—	0.54 **	0.02
5	Self-efficacy					—	−0.05
6	Radiation-related risk perception (2–8)						—

** *p* < 0.01. *^1^ Number of missing data: FPAM = 4, self-esteem = 1, self-efficacy = 8. *^2^ FPAM = Fukushima Future Parents Attitude Measure. *^3^ WHO-5 = World Health Organization-Five Well-Being Index.

**Table 4:** Logistic regression analysis of depressive symptoms in the negative and positive groups *^6^.

		Depressive Symptoms *^1^, OR *^7^ (95% CI)			
		Model 1 *^3^		Model 2 *^4^	
Living arrangement (reference: Living together)					
	Living alone	0.99	(0.51–1.92)	1.00	(0.51–1.94)
Age (reference: 18 years) *^5^					
	19	0.66	(0.27–1.60)	0.65	(0.27–1.59)
	20	0.88	(0.36–2.13)	0.88	(0.36–2.15)
	21	1.01	(0.40–2.57)	1.00	(0.39–2.60)
	22	0.27	(0.08–0.89)	0.28	(0.08–0.94)
Residence prefecture at the time of the Great East Japan Earthquake (reference: Other prefecture)					
	Fukushima prefectures	0.96	(0.50–1.81)	0.95	(0.50–1.81)
Perception of radiation risk					
	Delayed effects: High (reference: Low) *^2^	0.67	(0.34–1.33)	0.67	(0.34–1.35)
	Genetic effects: High (reference: Low) *^2^	1.15	(0.60–2.20)	1.14	(0.59–2.22)
The FPAM scale					
	Caring for a child (1-point increment)	1.16	(1.03–1.30)	1.13	(1.00–1.27)
	Giving birth to a baby (1-point increment)	1.12	(0.96–1.31)	1.10	(0.94–1.29)

*^1^ Participants in the positive group for depressive symptoms scored 1 or 0 for any of the 5 World Health Organization-Five Well-Being Index items or had an overall score of <13. *^2^ Scores of 3 or 4 on these scales are high, and scores of 1 or 2 are low. *^3^ Unadjusted and logistic regression analysis. *^4^ Adjusted Rosenberg Self-Esteem Scale and General Self-Efficacy Scale. *^5^ As there were only 3 participants aged ≥23 years, we excluded them from analysis. *^6^ Number of missing data: FPAM = 4, self-esteem = 1, self-efficacy = 8. *^7^ OR = Odds Ratio.
